# Bacteria in Solitary Confinement

**DOI:** 10.1128/JB.02509-14

**Published:** 2015-01-21

**Authors:** Conrad W. Mullineaux

**Affiliations:** School of Biological and Chemical Sciences, Queen Mary University of London, London, United Kingdom; Freiburg Institute for Advanced Studies (FRIAS), University of Freiburg, Freiburg, Germany

## Abstract

Even in clonal bacterial cultures, individual bacteria can show substantial stochastic variation, leading to pitfalls in the interpretation of data derived from millions of cells in a culture. In this issue of the *Journal of Bacteriology*, as part of their study on osmoadaptation in a cyanobacterium, Nanatani et al. describe employing an ingenious microfluidic device that gently cages individual cells (J Bacteriol 197:676–687, 2015, http://dx.doi.org/10.1128/JB.02276-14). The device is a welcome addition to the toolkit available to probe the responses of individual cells to environmental cues.

## BACTERIA ARE INDIVIDUALS!

Clonal bacterial cultures are often tacitly assumed to contain hundreds of millions of essentially identical cells. However, measurements on single bacterial cells show that bacteria often remain stubbornly individualistic, even when all possible measures have been taken to make the culture as uniform as possible. Such measures include recent propagation from a single cell, to ensure that all cells are genetically close to identical, which may be combined with careful culturing to ensure that all the bacteria experience the same environment and synchronization of the culture to put all cells at a similar stage in the cell cycle. These measures can deal with some of the causes of individual differences, but they cannot deal with the phenomenon of stochastic variation of gene expression, which can lead to strikingly different phenotypes even in genetically identical cells grown under identical conditions (1). Usually, these stochastic differences most probably have their origin in complex multistable signal transduction networks that settle into different patterns as a result of tiny changes in initial conditions (1, 2). Asymmetrical inheritance of cell structures is another way to generate diversity (3). For example, striking individual differences have been revealed by variations in the chemotactic behavior of individual Escherichia coli cells (4) and in the fluorescence signals from individual cells expressing a fluorescent protein (5). Phenotypic variability may be an important trick for bacterial survival in an unpredictable environment (6, 7). For example, the stochastic development of persister cells helps pathogens to survive antibiotic treatment, with clinical consequences (2).

## PITFALLS IN INFERRING THE PROPERTIES OF A BACTERIAL CELL FROM MEASUREMENTS ON A FLASK OF CELL CULTURE

A plethora of techniques in bacteriology rely on the measurement of some property of a clonal bacterial culture containing on the order of hundreds of millions of cells, simply because the measurement is not sensitive enough to reveal the characteristics of an individual cell. For example, spectroscopic measurements generally report on the interaction of a light beam with a cuvette of cell suspension, perhaps leading to inferences about the cellular content of a particular protein complex or giving dynamic information about a particular physiological process. Biochemical techniques like SDS-PAGE and immunoblotting reveal the composition of a protein extract from a substantial volume of cell culture. Microarrays and RNA deep sequencing analyze the properties of RNA extracted from cell cultures, and proteomic and metabolomic techniques similarly reveal the composition of protein and small-molecule extracts from flasks of cell culture. If the ultimate aim is to reveal the workings of the bacterial cell, there could be pitfalls in interpreting such data. To give one example, metabolomic data can be used to feed into integrated models of bacterial metabolism, on the assumption that the metabolites extracted from the cell culture reveal the population of small molecules in each cell in the culture. However, if the cells in the culture actually display a range of phenotypes, then the averaged phenotype derived from the bulk measurements could be quite distinct from any of the phenotypes displayed by individual cells in the culture (7). This could lead to an erroneous picture of the metabolic network in the cell.

In their study on the roles of potassium transporters in osmoadaptation in a cyanobacterium (8), Nanatani et al. grappled with a different problem that also has its origin in individual differences. These authors needed to monitor volume changes in individual cells of a cyanobacterium following hyperosmotic shock (8). The diameter of a single cell is relatively easy to measure, and it would be straightforward to measure the diameters of a population of cells before and after osmotic shock, either by microscopy or by flow cytometry, for example (9, 10). However, in a population of cells with a range of cell sizes and possibly showing various responses to the osmotic shock, it is much more powerful to look at the same individual cells before and after applying the shock and at the kinetics of volume changes following the shock. This is much harder to achieve. The fates of individual cells can be followed in the microscope when they are adsorbed onto agar or trapped under a coverslip, but these methods do not permit the medium to be changed during the measurement, which is clearly a requirement in this case. Bacteria can sometimes be fixed onto glass slides with polylysine and the medium changed by flushing the liquid through between the slide and the coverslip (see reference 11 for an example). However, polylysine does not work well with all bacteria, and the fixation and change of medium can cause unwelcome mechanical stress to the cells. Therefore, Nanatani et al. developed a different solution (8).

## A NEW METHOD TO TRAP AND STUDY SINGLE BACTERIAL CELLS

In order to trap individual cells and study their responses to hyperosmotic shock, Nanatani et al. developed a microfluidic device that cages single cells between hydrogel walls (8). The hydrogel walls are water permeable, allowing the medium to be replaced without flushing away the cells under observation or mechanically stressing them. The same principle has previously been used for caging eukaryotic cells (12–15), but Nanatani et al. produced much smaller cages suitable for confining bacteria; in this case, spherical cells with a diameter of 2 to 3 μm. The technique proved highly successful for microscopic measurement of the changes in volume of individual cells following hyperosmotic shock (8), but this is only one possible application of the method. The technique could open the door to real-time studies of single-cell responses to many other kinds of stress that could be imposed by changing the medium or to specific chemicals and signaling molecules. Such studies have long been practiced in eukaryotic cells, facilitated by larger size and (in some cases) the ability of the cells to tightly adhere to surfaces. See reference 16 for an early example. The microfluidic device developed by Nanatani et al. (8) should make it easier to try the same things in bacteria, potentially giving bacteriologists a whole new window on single-cell behavior.

## References

[1] Avery SV 2006 Microbial cell individuality and the underlying sources of heterogeneity. Nat Rev Microbiol 4:577–587. doi:10.1038/nrmicro1460. 16845428

[2] Davidson CJ, Surette MG 2008 Individuality in bacteria. Annu Rev Genet 42:253–268. doi:10.1146/annurev.genet.42.110807.091601. 18652543

[3] Kulasekara BR, Kamischke C, Kulasekara HD, Christen M, Wiggins PA, Miller SI 2013 c-di-GMP heterogeneity is generated by the chemotaxis machinery to regulate flagellar motility. Elife 2:e01402. doi:10.7554/eLife.01402. 24347546 PMC3861689

[4] Sourjik V, Wingreen NS 2012 Responding to chemical gradients: bacterial chemotaxis. Curr Op Cell Biol 24:262–268. doi:10.1016/j.ceb.2011.11.008. 22169400 PMC3320702

[5] Raj A, van Oudenaarden A 2008 Nature, nurture or chance: stochastic gene expression and its consequences. Cell 135:216–226. doi:10.1016/j.cell.2008.09.050. 18957198 PMC3118044

[6] Veening J-W, Smits WK, Kuipers OP 2008 Bistability and bet-hedging in bacteria. Annu Rev Microbiol 62:193–210. doi:10.1146/annurev.micro.62.081307.163002. 18537474

[7] Lidstrom ME, Konopka MC 2010 The role of physiological heterogeneity in microbial population behaviour. Nat Chem Biol 6:705–712. doi:10.1038/nchembio.436. 20852608

[8] Nanatani K, Shijuku T, Takano Y, Zulkifli L, Yamazaki T, Tominaga A, Souma S, Onai K, Morishita M, Ishiura M, Hagemann M, Suzuki I, Maruyama H, Arai F, Uozumi N 2015 Comparative analysis of *kdp* and *ktr* mutants reveals distinct roles of the potassium transporters in the model cyanobacterium Synechocystis sp. strain PCC 6803. J Bacteriol 197:676–687. doi:10.1128/JB.02276-14. 25313394 PMC4334184

[9] Müller S, Nebe-von-Caron G 2010 Functional single-cell analyses: flow cytometry and cell sorting of microbial populations and communities. FEMS Microbiol Rev 34:554–587. doi:10.1111/j.1574-6976.2010.00214.x. 20337722

[10] Shapiro HM 2000 Microbial analysis at the single-cell level: tasks and techniques. J Microbiol Methods 42:3–16. doi:10.1016/S0167-7012(00)00167-6. 11000426

[11] Tipping MJ, Steel BC, Delalez NJ, Berry RM, Armitage JP 2013 Quantification of flagellar motor stator dynamics through *in vivo* proton-motive force control. Mol Microbiol 87:338–347. doi:10.1111/mmi.12098. 23216828

[12] Wakamoto Y, Ramsden J, Yasuda K 2005 Single-cell growth and division dynamics showing epigenetic correlations. Analyst 130:311–317. doi:10.1039/b409860a. 15724159

[13] Peng CC, Liao WH, Chen YH, Wu CY, Tung YC 2013 A microfluidic cell culture array with various oxygen tensions. Lab Chip 13:3239–3245. doi:10.1039/c3lc50388g. 23784347

[14] Gomez-Sjoberg R, Leyrat AA, Pirone DM, Chen CS, Quake SR 2007 Versatile, fully-automated, microfluidic cell culture system. Anal Chem 79:8557–8563. doi:10.1021/ac071311w. 17953452

[15] Kim MJ, Lee SC, Pal S, Han E, Song JM 2011 High-content screening of drug-induced cardiotoxicity using quantitative single cell imaging cytometry on microfluidic device. Lab Chip 11:104–114. doi:10.1039/c0lc00110d. 21060932

[16] Woods NM, Cuthbertson KSR, Cobbold PH 1986 Repetitive transient rises in cytoplasmic free calcium in hormone-stimulated hepatocytes. Nature 319:600–602. doi:10.1038/319600a0. 3945348

